# Evaluation of Dietary Intake in Individuals with Mild Cognitive Impairment

**DOI:** 10.3390/nu15173694

**Published:** 2023-08-23

**Authors:** Shih-Wei Nien, I-Hsin Lin, Hsiu-Chuan Wu, Yi-Hsiu Chen, Suh-Ching Yang

**Affiliations:** 1 School of Nutrition and Health Sciences, Taipei Medical University, Taipei 11031, Taiwan; nina0904@cgmh.org.tw (S.-W.N.); ma07108007@tmu.edu.tw (Y.-H.C.); 2 Department of Medical Nutrition Therapy, Linkou Chang Gung Memorial Hospital, Taoyuan 333423, Taiwan; cabbage@cgmh.org.tw; 3 Department of Neurology, Linkou Chang Gung Memorial Hospital, Taoyuan 333423, Taiwan; serenawu@cgmh.org.tw; 4 Research Center of Geriatric Nutrition, College of Nutrition, Taipei Medical University, Taipei 11031, Taiwan; 5 Nutrition Research Center, Taipei Medical University Hospital, Taipei 11031, Taiwan; 6 School of Gerontology and Long-Term Care, College of Nursing, Taipei Medical University, Taipei 11031, Taiwan

**Keywords:** mild cognitive impairment, dietary intake, dietary pattern, MIND diet

## Abstract

The phase of mild cognitive impairment (MCI) holds significant importance for postponing the onset of dementia. Therefore, MCI has become a central focus in research related to dementia prevention. The purpose of this study was to investigate the dietary intake and dietary patterns of MCI patients in Taiwan. In total, 40 subjects were enrolled in this cross-sectional study that was conducted from July 2019 to September 2021 at the Linkou Chang Gung Memorial Hospital. The results of the clinical dementia rating (CDR) and mini-mental state examination (MMSE) were obtained from medical records. Participants were divided into two groups: a healthy group (MMSE ≥ 26 points, CDR = 0) and an MCI group (MMSE ≥ 26 points, CDR = 0.5). Results indicated that the MCI group had significantly higher copper and lower low-fat meat intake compared to the healthy group. Furthermore, the high MIND (Mediterranean dietary approaches to stop hypertension intervention for neurodegenerative delay) diet score represented a lower risk of MCI. After adjusting for age, gender, diabetes mellitus, hypertension, and calorie intake in the multivariate regression analysis, calcium and fruit intake levels were positively associated with the MMSE, whereas low-fat meat intake was negatively associated with the CDR. In conclusion, the prevalence of MCI demonstrated a close correlation with nutrient intake, including copper and calcium. Furthermore, a MIND diet, particularly one high in n-3 polyunsaturated fatty acids, might be useful for preventing MCI. However, more extensive research with larger populations is needed to confirm this potential.

## 1. Introduction

Along with the aging of the population, the incidences of age-related diseases, especially dementia, continue to rise. According to the International Dementia Association’s 2019 Global Dementia Report and previous studies, it was estimated that there were more than 50 million people with dementia worldwide, which was expected to grow to 152 million by 2050 [1]. According to an epidemiological survey of dementia conducted by the Taiwan Dementia Association in 2019, there were approximately 3.6 million (7.78%) people over the age of 65 years in Taiwan [2]. Of these, about 0.65 million had mild cognitive impairment (MCI). Studies showed that an MCI diagnosis in a healthy older person represents a transition period before the onset of dementia and that about 15~35% of them will progress to dementia each year [3]. As there is currently no effective way to prevent or treat dementia, delaying the progression of MCI to dementia is a potential way to reduce the incidence of dementia.

Several studies have indicated that reducing dementia risk factors such as hypertension, diabetes mellitus (DM), and cardiovascular disease (CVD) can prevent its progression [4,5]. Therefore, dietary recommendations, including dietary intake and dietary patterns, for delaying dementia have been evaluated in recent years [6,7,8,9]. The MIND (Mediterranean dietary approaches to stop hypertension (DASH) intervention for neurodegenerative delay) diet emphasizes dietary components and servings linked to neuroprotection and dementia prevention [10,11]. The MIND diet emphasizes eating more natural plant foods such as green leafy vegetables, colored vegetables (including bright red, yellow, and green vegetables), nuts, berries, legumes and whole grains, poultry, olive oil, and wine, and limiting the intake of saturated fat-rich foods such as red meat, marinara, margarine, cheese, pastries and fast foods, sweets, and candy [10]. The MIND diet contains foods rich in certain vitamins, carotenoids, and flavonoids that are believed to protect the brain by reducing oxidative stress and inflammation [8]. The MIND diet score, which is calculated by dietary components, including 10 brain-healthy foods and 5 unhealthy foods, was associated with a slower rate of cognitive decline equivalent to 7.5 years of younger age among participants in a high-score group compared to a low-score group [7,8]. A previous study also reported that the MIND diet score was positively related to verbal memory scores in a 12.9-year follow-up study [9]. Although individuals in subtropical and tropical countries consume vegetables and dark-color vegetables such as onions, garlic, tomatoes, mushrooms, bell pepper, and eggplants, some of the foods recommended in the MIND diet are not easily available in markets, including berries and wine. Therefore, there is an urgent need to understand and collect data on the dietary intake of patients with MCI in a tropical or subtropical country facing an increasing prevalence of dementia due to an aging society.

**Figure 1:**
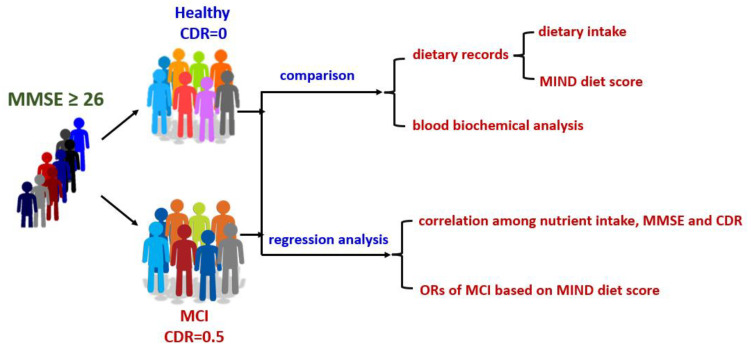
Scheme of the study. MMSE, mini-mental state examination; CDR, clinical dementia rating; PUFAs, polyunsaturated fatty acids; MIND, Mediterranean dietary approaches to stop hypertension intervention for neurodegenerative delay; ORs, odd ratios; MCI, mild cognitive impairment.

In this study, dietary patterns and intake, as well as relative associations, were evaluated in Taiwanese patients with MCI (Figure 1). The novelty of this study was the calculation of MIND diet scores based on a subtropical-style diet.

## 2. Materials and Methods

### 2.1. Study Design and Participants

A cross-sectional study was conducted between July 2019 and September 2021 at Linkou Chang Gung Memorial Hospital (Taoyuan, Taiwan). Both MMSE and CDR serve as assessment tools for diagnosing cognitive function impairment [12,13]. In this study, MCI was defined as MMSE scores ≥ 26 points and CDR scores of 0.5 points. Mild to moderate dementia was categorized as MMSE scores of 10–26 points and CDR scores ranging from 1 to 2 points. Moderate to severe dementia was determined using MMSE scores of 10–14 points or CDR scores of 2 points, while severe dementia was indicated by MMSE scores of 5–9 points and CDR scores of 3 points. We included patients who had completed 3 days of dietary records (two weekdays and one holiday), a brief mental state examination (MMSE ≥ 26), clinical dementia scale (CDR = 0 or CDR = 0.5), and independent activities. The MMSE and CDR were conducted by a psychiatrist. Subjects were excluded if they had extreme dietary analytical results (<500 kcal or >3500 kcal), no caregiver, an inability to speak clearly, a malignant tumor, or a history of stroke or dementia. The study protocol was approved by the Chang Gung Medical Foundation’s Institutional Review Board (no. 201900610B0 on 29 April 2019) and was registered with the Clinical Trials Protocol Registration and Results System (PRS) for approval (NCT05028920).

### 2.2. Data Collection of Dietary Intake

Participants’ characteristics, which were obtained from medical records, included age, sex, body height, body weight, smoking, drinking, educational level, comorbidities (diabetes; hypertension; and history of cardiovascular disease, stroke, and cerebrovascular disease), blood pressure, and types of drugs used. The body mass index (BMI) was calculated as weight in kilograms divided by the square of height in meters. Waist circumference (WC) in centimeters was noted, and body composition was assessed using InBody S10 (InBody S10; Biospace, Seoul, the Republic of Korea) via bioelectrical impedance analysis.

Participants completed dietary records over 3 days (2 weekdays and 1 weekend) before visiting a dietitian for the latest follow-up session. To confirm dietary records, 24 h dietary recall was completed through face-to-face interviews with a well-trained dietitian. Dietary energy and nutrient intake were calculated using nutrition analysis software (Cofit Pro vers. 1.0.0, Cofit HealthCare, Taipei, Taiwan) [14]. Macronutrient and micronutrient intake levels were calculated according to guidelines presented in the database of the Taiwan Ministry of Health and Welfare (MOHW) [15]. The MIND diet score was calculated based on Morris et al. [8]. According to contents of the MIND diet, dietary records were converted into daily or weekly intake times or servings and then scored based on MIND diet suggestions (each item was awarded 0, 0.5, or 1 points). The MIND diet score consists of 15 dietary components, including 10 brain-healthy food groups (green leafy vegetables, other vegetables, nuts, berries, soybeans, whole grains, fish, poultry, olive oil, and wine) and 5 unhealthy food groups (red meats, butter and stick margarine, cheese, pastries and sweets, and fried/fast food). The consumption of olive oil was given a score of 1 if the participant indicated that this was the main oil usually used at home; otherwise, a score of 0 was given. For all other diet components, a concordance score of 0, 0.5, or 1 was assigned by summing the frequency of consumption of each food portion associated with that component. All 15 component scores were summed to give the total MIND diet score. The scoring scale and converted servings of USA and Taiwan are shown in Supplementary Table S1.

### 2.3. Measurement Items

Blood was collected from participants after a 12 h fast and sent to the Laboratory Medicine Department of Chang Gung Memorial Hospital (Linkou, Taiwan) for biochemical analyses. The liver function index (aspartate aminotransferase, AST; and alanine aminotransferase, ALT), lipid profile (total cholesterol, TC; triglycerides, TGs; high-density lipoprotein cholesterol, HDL-C; and low-density lipoprotein cholesterol, LDL-C), kidney function indicators (blood urea nitrogen, BUN; and creatinine, Cr), glucose-related indicators (fasting plasma glucose, FPG; glycated hemoglobin, HbA1c), and the albumin level were measured via the ADVIA 1800^®^ Clinical Chemistry System (Siemens Healthcare, Erlangen, Germany). Blood folate and vitamin B_12_ levels were measured using a Roche Cobas^®^ e411 ElectroChemiLuminescence (ECL) immunoassay analyzer (Roche, Basel, Switzerland).

### 2.4. Statistical Analysis

Values are presented as mean ± standard deviation (SD) or percentage (%). The Shapiro–Wilk test was used to test the normality of the population. Comparisons of values between cognitive impairment and nutrition intake were tested using a *t*-test or Wilcoxon signed-rank test. A Chi-squared test was performed for categorical variables, such as basic information, body composition, and dietary intake. Univariate linear regression was used to test correlations among nutrient intake, dietary intake, MMSE, and CDR. Further, variables with statistical differences were analyzed using multivariate regression. A logistic regression was used to analyze the odds ratio (OR) of MCI by setting the MIND diet score of <7.5 as a reference. The possible affecting factors of MCI, such as age, sex, DM, hypertension, and calorie intake, were adjusted according to previous publications [4,5]. Statistical analyses were performed using SAS software 9.4 (SAS institute, Cary, NC, USA). A *p* value of <0.05 was defined as a statistically significant difference.

## 3. Results

### 3.1. Characteristics of Participants

A total of 50 participants were recruited in this study, but 10 participants were excluded due to unmatched data. Thus, 40 participants entered this study, 19 in the healthy group (MMSE ≥ 26 points, CDR = 0) and 21 in the MCI group (MMSE ≥ 26 points, CDR = 0.5). As shown in Table 1, 16 participants were overweight (40.0%), 9 participants were obese (22.5%), 13 participants had diabetes (32.5%), 21 participants had hypertension (52.5%), and 12 participants had hyperlipidemia (30.0%). When comparing differences between the healthy and MCI groups, the MCI group had significantly higher CDR scores than the healthy group.

### 3.2. Nutrient Intake

According to the 3-day dietary record, the MCI group had a significantly higher intake of copper than the healthy group (Table 2). There was no difference in other nutrients between the two groups. Compared to the 8th version of dietary reference intakes (DRIs) of Taiwan, both the healthy and MCI groups had lower carbohydrate, monounsaturated fatty acid (MUFA), fiber, vitamin B_6_, niacin, calcium, and zinc intake. Furthermore, protein, fatty acid, saturated fatty acid (SFA), cholesterol, vitamin A, vitamin C, vitamin B_12_, and phosphorus intake levels were higher than the DRIs in both groups. Moreover, polyunsaturated fatty acid (PUFA) and iron intake levels were less than the DRIs in the MCI group, while PUFA and iron intake were higher than the DRIs in the healthy group (Table 2). Vitamin E intake was less than the DRI in the healthy group, whereas vitamin E intake was higher than the DRI in the MCI group.

### 3.3. Dietary Intake

Compared to the healthy group, the MCI group had a significantly lower intake of low-fat meat and proteins (Table 3).

### 3.4. MIND Diet Score

There was no difference in the MIND diet scores between the healthy and MCI groups (Table 4).

### 3.5. Regression Analysis

#### 3.5.1. Nutrient Intake and Cognitive Function

After adjusting for sex and age, the nutrient intake levels and MMSE scores were analyzed using univariate linear regression analysis. The results showed that vitamin A (β < 0.001, *p* = 0.02), vitamin C (β = 0.01, *p* = 0.008), and calcium (β < 0.001, *p* = 0.004) were positively associated with the MMSE score. The results of the multivariate linear regression analysis revealed that calcium (β = 0.001, *p* = 0.04) was positively associated with the MMSE score (Table 5).

The univariate linear regression analysis indicated that n-3 PUFAs (β = −0.06, *p* = 0.04) were negatively associated with the CDR score, while copper (β < 0.001, *p* = 0.02) was positively associated with the CDR score. The results of the multivariate linear regression analysis indicated that copper (β = 0.001, *p* = 0.02) was positively associated with the CDR score (Table 5).

#### 3.5.2. Dietary Intake and Cognitive Function

After adjusting for sex and age, the results showed that fruits positively predicted the MMSE score (β = 0.76, *p* = 0.01; β = 0.81, *p* = 0.01). Moreover, low-fat meat and protein intake were negatively associated with the CDR score (β = −0.08, *p* = 0.01) (Table 6).

#### 3.5.3. MIND Diet Score and Cognitive Function

MIND diet scores were calculated based on a 3-day dietary record, and 7.5 was set as the cutoff point for high and low MIND diet scores. As a result, the average score was 9.2 (*n* = 25) in the high-MIND diet group, while the low-MIND diet score group was 6.9 (*n* = 15). The results also showed that the high-score group had a significantly lower risk of MCI compared to the low-score group (OR = 0.23, 95% CI = 0.06~0.99, *p* = 0.04) (Table 7).

### 3.6. Blood Biochemical Parameters

The average FPG and HbA1C values in all participants were slightly higher than the normal range, at 104.6 ± 26.3 mg/dL and 6.2 ± 0.9%, respectively. Blood lipid profiles were all within normal levels. There were no differences in biochemical parameters between the two groups (Table 8).

## 4. Discussion

### 4.1. Basic Characteristics of Participants

There were no differences in the basic characteristics between the two groups (Table 1). Hypertension is a risk factor for MCI. A prospective study of women aged 65 to 79 years who were followed up for 9.1 years indicated that hypertension would increase the risk of MCI by 20% [16]. That study also reported that participants with a blood pressure of 140/90 mmHg had a 30% increased risk of MCI, regardless of whether or not they were taking antihypertensive drugs [16]. The possible mechanism for this result might be related to hypertension increasing the risk of ischemic white matter lesions in the brain and then causing cognitive impairment [17]. Liao et al. reported that people with high blood pressure were up to 2.3 times more likely to have lesions in the white matter of the brain [18]. In this study, it was found that the MCI group had a trend of a higher SBP (140.9 ± 15 mmHg) (Table 1). More participants should be enrolled in future studies to clarify the relationship between hypertension and MCI.

### 4.2. Nutrients and Cognitive Impairment

There was no difference in caloric intake between the two groups (Table 2). No association was observed between energy intake and MMSE or energy intake and CDR (Table 5). Previous studies have reported that excessive caloric intake could increase oxidative stress injury and β-amyloid accumulation, which harms nerve cells and increases the risk of MCI [19,20]. However, an obviously high caloric intake was not observed in this study.

In addition, there was no difference in carbohydrate intake between the two groups (Table 2). No association was found between carbohydrate intake and the MMSE or between carbohydrate intake and the CDR (Table 5). Excessive carbohydrate intake was positively associated with cognitive impairment and dementia in an earlier study [21]. A cohort study reported that after screening 937 participants with CDR, 200 participants had MCI, and it was found that the 4th quartile (with carbohydrate intake of 299 g, comprising 58% of the total calorie intake) had a 1.89-fold MCI or dementia risk compared to the 1st quartile (with a carbohydrate intake of 172 g, comprising 47% of the total calorie intake) [22]. The possible mechanism for this result might be high blood sugar or diabetes, which can induce the synthesis of advanced glycation end products (AGEs) and increase oxidative stress [23,24] and further result in cognitive impairment and amyloidosis [23,24]. In this study, the carbohydrate intake of the MCI group was much lower than in the previous cohort study [22]. This might be related to the cognitive impairment stages (mild to severe), differences in the food assessment tools (food frequency questionnaire and 3-day food record), different dietary styles (Eastern vs. Western), or the sample size of recruited participants.

In this study, we did not find a difference in protein intake between the two groups (Table 2). Furthermore, protein intake vs. MMSE and protein intake vs. CDR scores were not associated (Table 5). This result was similar to a previous study by Robert et al. [22]. A protein supplementation study also indicated that protein supplementation (a 15 g protein drink twice a day) might improve reaction time performance in pre-frail and frail elderly but did not improve other cognitive functions [25]. However, a Korean study of MMSE and protein intake reported that MCI participants had a lower protein intake than healthy women [26]. After a correlation analysis, it was found that the MMSE score was positively correlated with protein intake, which would decrease neurotransmitter amino acid synthesis, such as tryptophan [27]. Therefore, the relationship between protein intake and cognitive function remains controversial and needs to be further investigated.

An excessive intake of SFAs will increase the risk of MCI and dementia; conversely, an increase in dietary unsaturated fatty acids can reduce the risk of cognitive impairment [25]. Morris et al. collected food frequency questionnaires from 815 participants over 65 years of age and found that there were negative correlations between n-3 PUFA intake and the risks of MCI and dementia [28]. The possible mechanism was that n-3 PUFAs could regulate the anti-inflammatory response and endothelial function through cyclooxygenase (COX) and lipoxygenase (LOX) and slow down cognitive decline [29,30,31]. However, an intervention study with n-3 PUFA supplementation for 6 months indicated that n-3 PUFA supplementation did not palliate cognitive decline [32]. Although the intake of total fat of MUFAs, PUFAs, and SFAs did not show differences between the two groups (Table 2), the n-3 PUFA intake was negatively associated with the CDR score (Table 5) in this study. Taken together, further research is needed to clarify how the consumption of fatty acids affects cognitive function and the relationship between PUFAs and cognitive function.

Regarding vitamin intake, no differences were found between the two groups (Table 2); however, vitamin A and C intake could positively predict MMSE scores in the univariate linear regression analysis (Table 5). Previous studies reported that there was no correlation between CDR scores and dietary vitamin A or E [21,33]. Moreover, vitamin C intake showed a positive relationship with MMSE scores in a correlation analysis [33]. A previous study demonstrated that vitamins C and E are antioxidative reagents and can activate superoxide dismutase (SOD) and plasma glutathione peroxidase (GPx), thereby reducing oxidative stress and the risk of cognitive impairment [33]. Therefore, the preventive effect of vitamin C on cognitive impairment was predictable.

No differences were seen in the intake of vitamin B_6_, B_12_, and folate between the two groups when comparing the two groups using a *t*-test or linear regression analysis (Table 5). It is known that vitamin B_6_, B_12_, and folate participate in the metabolism of homocysteine, which is positively related to cognitive impairment and dementia [32]. Thus, insufficient intake of B_6_, B_12_, and folate may disturb the metabolism of homocysteine into methionine and cystathionine, which can cause DNA damage and impair the transmission of neurotransmitters [34]. However, Butler et al. reported that vitamin B complex supplementation could not prevent MCI [35]. A randomized control trial also indicated that when older participants with MCI supplemented with B_12_ (500 µg) and folate (400 µg) daily for 24 months, the average CDR score remained unchanged [36]. As a result, the effects of vitamin B supplementation on preventing MCI remain controversial.

Most mineral intake levels showed no difference between the healthy and MCI groups, except for copper (Table 2). After a univariate linear regression or multivariate linear regression analysis, copper intake was positively associated with CDR scores (Table 5). Moreover, calcium intake was positively associated with MMSE scores (Table 5). A cross-sectional study showed that comparing the fourth quartile to the first quartile of copper intake, the adjusted OR was 0.34 for low cognitive performance [35]. Li et al. reported that excessive copper intake could increase ceruloplasmin and raise oxidative stress, which led to a lowering of cognitive performance [37]. It was reported that high dietary copper intake (10 mg/kJ) would increase forgetfulness by 1.25-fold in elderly women [38]. A previous prospective study showed that a high calcium and vitamin D intake (1200 mg/day and 800 IU/day) resulted in better cognitive performance than the low-dosage group (600 mg/day and 400 IU/day) [39]. Further studies or interventions are needed to prove the relationship between mineral intake and MCI, especially copper and calcium.

### 4.3. Food Categories and Cognitive Function

The MCI group had significantly lower soybean and low-fat meat intake (Table 3), which was negatively associated with CDR scores (Table 6). Two systematic reviews reported that protein intake was not highly related to cognitive impairment, but the source of protein might be related to the risk of cognitive impairment [40,41]. Research from Singapore reported that a high red meat intake was positively associated with a high risk of cognitive impairment, whereas a high fresh fish and seafood intake was associated with a lower risk of cognitive impairment [42]. Fish intake is negatively associated with cognitive impairment, or dementia might be related to the high content of n-3 PUFAs [41]. Therefore, it was suggested that soybeans and low-fat meat might delay the progression of MCI.

This study found that fruit intake was positively associated with MMSE scores (Table 6). Previous studies reported that vegetables and fruits are rich in antioxidant nutrients and phytochemicals, such as β-carotene, vitamin C, vitamin E, folic acid, flavonoids, and polyphenols, which can inhibit neurodegeneration caused by oxidative stress and can produce a lower risk of dementia [43,44,45]. A previous systemic review pointed out that increasing fruit and vegetable intake was related to a reduced risk of MCI [46]. Another study indicated that those who had more than two servings of vegetables and fruits had higher MMSE scores than those who had less than one serving of vegetables and fruits [26]. Thus, further studies could focus on the effects of different fruits and vegetables on cognitive performance.

### 4.4. MIND Diet and Cognitive Function

In this study, it was found that subjects with higher MIND diet scores had lower ORs of MCI (Table 7), although the MIND diet score did not significantly differ between the two groups (Table 4). Higher adherence to the MIND diet led to lower incidences of cognitive impairment, dementia, and Parkinson’s disease [7,8,33,47]. MIND diet guidelines have similar contents to the Mediterranean diet and DASH diet, including plant-based foods, dietary fiber, and low-fat meat, which can protect the brain from neurodegeneration and prevent cognitive impairment [48,49,50,51]. Malek Rivan et al. demonstrated that high tropical fruits–oats dietary patterns protected against dementia in older adults [52]. The possible reason that the MIND diet can reduce the MCI risk is that the MIND diet includes more green leafy vegetables and berries which are rich in lutein, vitamin K (phylloquinone), folic acid, α-tocopherol, β-carotene, flavonoids, tannins, phenolic acids, and lignans [53]. These vitamins and phytochemicals can reduce phospholipid peroxidation and inhibit neuron inflammation, tau protein phosphorylation, and β-amyloid deposition, which result in less injury to memory [53]. On the other hand, the MIND diet also emphasizes foods that induce inflammation and oxidative stress, such as cakes, sweets, fried foods, and margarine, and are high in sugar, SFAs, and trans-fatty acids, which are not conducive to brain health [8]. Shakersain et al. reported that participants with higher MIND diet scores presented a lower risk of cognitive impairment (MMSE score ≤ 24) [54]. Based on the results of this study, the MIND diet score may be a useful predictor for dietary patterns of cognitive decline in Asian countries.

### 4.5. Body Composition and Blood Biochemical Parameters

There were no differences in the body composition between the two groups; however, participants were defined as overweight (>24 kg/m^2^) according to the definition used in Taiwan (Table 8). A longitudinal study that analyzed over 7000 participants’ body composition and cognitive function found that participants with a BMI of <20 kg/m^2^ had lower memory function scores, which could be a determinant of cognitive decline in the elderly. A possible reason for this result is that body weight loss is a symptom of pre-dementia and might be related to some psychological symptoms such as loss of interest in daily life and being indifferent to others [55]. However, overweight or obese people have a higher risk of MCI due to chronic inflammation, CVDs, DM, or hypertension, which are related to vascular risk factors [19,56].

No difference was found in the blood biochemical assessment between the two groups in this study (Table 8). Previous studies reported that MCI participants had higher fasting blood glucose, post-meal blood glucose, and HbA1c compared to healthy participants and had a higher risk of neuropathy-induced cognitive impairment [57,58]. In this study, the MCI group had higher FPG, which was defined as impaired glucose intolerance (≥100 and <126 mg/dL). Therefore, continuous monitoring of blood glucose levels is necessary to control the progression of MCI. 

### 4.6. Strengths and Limitations

This is the first study to analyze the relationships between dietary patterns and nutrient intake in an MCI population in Taiwan. This study also confirmed whether or not the MIND diet score can be used to assess Asian diets. However, there are still some limitations in this study. First, this was a cross-sectional study and only enrolled participants in northern Taiwan. Second, the small sample size could not represent the entire MCI population and could not clarify causal relationships. Based on setting α at 0.05, the power value was 0.803 when calculated using a low-fat meat intake and 0.42 when calculated using the MIND diet score (G*Power software version 3.1 (Heinrich-Heine-Universität Düsseldorf, Düsseldorf, Germany). Third, only the MMSE and CDR were used for the cognitive impairment assessment. Other assessment tools should be added in future studies. Lastly, follow-up is necessary for longitudinal observation of the influence of dietary patterns on cognitive impairment.

## 5. Conclusions

This study compared dietary patterns and nutrient intake between healthy (MMSE ≥ 26, CDR = 0) and MCI (MMSE ≥ 26, CDR = 0.5) subjects and indicated that participants with MCI had higher copper and lower low-fat meat intake. Furthermore, vitamins A and C and calcium were positively associated with MMSE scores, whereas n-3 PUFAs were negatively associated with CDR scores. In addition, subjects with high MIND diet scores had lower ORs for MCI. According to the current findings, a MIND diet, particularly a diet low in saturated fat and high in n-3 polyunsaturated fatty acids, might be useful for preventing MCI. However, it is necessary to continuously demonstrate this suggestion with a larger population.

## Figures and Tables

**Table 1:** Characteristics of participants ^1,2,3^.

Characteristic	All (*n* = 40)	Healthy (*n* = 19)	MCI (*n* = 21)	*p*
Age, years	71.4 ± 6.6	72.4 ± 7.0	70.5 ± 6.3	0.42
Male, *n* (%)	15 (37.5%)	6 (31.6%)	9 (42.9%)	0.48
Female, *n* (%)	25 (62.5%)	13 (68.4%)	12 (57.1%)	0.48
SBP, mmHg	139.5 ± 19.3	137.9 ± 23.5	140.9 ± 15	0.61
DBP, mmHg	77.3 ± 12.7	78.9 ± 12.5	75.8 ± 13	0.47
MMSE	27.5 ± 1.7	27.7 ± 1.9	27.3 ± 1.5	0.32
CDR	0.3 ± 0.3	0.0 ± 0.0	0.5 ± 0.0	<0.0001 *
Overweight, *n* (%)	16 (40.0%)	7 (36.8%)	9 (42.9%)	0.71
Obesity, *n* (%)	9 (22.5%)	5 (26.3%)	4 (19.0%)	0.60
DM, *n* (%)	13 (32.5%)	5 (26.3%)	8 (38.1%)	0.44
HTN, *n* (%)	21 (52.5%)	11 (57.9%)	10 (47.6%)	0.53
Hyperlipidemia, *n* (%)	12 (30.0%)	5 (26.3%)	7 (33.3%)	0.65

^1^ MCI, mild cognitive impairment; SBP, systolic blood pressure; DBP, diastolic blood pressure; DM, diabetes mellitus; HTN, hypertension; MMSE, mini-mental state examination; CDR, clinical dementia rating scale. ^2^ Data are expressed as the mean ± standard deviation. ^3^ Statistical analyses of data were conducted using Student’s *t*-test, Wilcoxon rank-sum test, and Chi-squared test. An asterisk (*) indicates a significant difference compared to the healthy group (*p* < 0.05).

**Table 2:** Nutrient intake in each group ^1,2,3^.

Nutrients	Healthy (n = 19)	MCI (*n* = 21)	*p*
Energy, kcal	1678.7 ± 305.2	1695.8 ± 278.3	0.72
CHO, g	188.6 ± 34.1	194.5 ± 33.4	0.59
CHO, %	45.4 ± 7.2	46.5 ± 6.3	
Protein, g	70.3 ± 16.4	70.1 ± 13.2	1.00
Protein, %	16.8 ± 2.6	16.5 ± 2.1	
Fat, g	72.5 ± 22.9	71.3 ± 22.0	0.72
Fat, %	38.3 ± 7.6	37.2 ± 7.1	
MUFAs, g	27.6 ± 9.8	28.1 ± 11.7	0.91
MUFAs, %	14.5 ± 3.2	14.9 ± 4.8	
PUFAs, g	23.0 ± 10.3	19.2 ± 8.2	0.18
PUFAs, %	12.2 ± 4.8	9.7 ± 2.7	
n-3 FAs, g	3.1 ± 1.6	2.2 ± 1.1	0.09
n-6 FAs, g	19.9 ± 9.3	17.0 ± 8.6	0.22
SFAs, g	19.6 ± 7.0	20.8 ± 6.8	0.61
SFAs, %	10.4 ± 2.7	10.8 ± 2.4	
Cholesterol, mg	249.6 ± 108.0	321.8 ± 138.3	0.10
Fiber, g	13.1 ± 6.8	14.2 ± 3.8	0.18
Vitamin A, μg	730.1 ± 343.8	962.4 ± 502.2	0.13
Vitamin E, mg	9.4 ± 7.1	18.9 ± 24.6	0.29
Vitamin C, mg	110.9 ± 64.2	110.8 ± 45.2	0.66
Vitamin B_1_, mg	1.1 ± 0.4	1.3 ± 0.6	0.37
Vitamin B_2_, mg	1.0 ± 0.3	1.0 ± 0.3	0.75
Niacin, mg	13.1 ± 3.7	14.2 ± 3.4	0.18
Vitamin B_6_, mg	1.5 ± 0.5	1.5 ± 0.4	0.63
Vitamin B_12_, μg	6.8 ± 10.3	3.0 ± 1.2	0.55
Folic acid, μg	250.6 ± 112.6	280.8 ± 82.5	0.18
Sodium, mg	1116.9 ± 935.0	832.3 ± 411.9	0.23
Potassium, mg	2151.6 ± 999.5	2089.0 ± 434.9	0.53
Calcium, mg	543.7 ± 415.5	508.5 ± 185.8	0.65
Magnesium, mg	261.9 ± 137.8	249.8 ± 63.5	0.42
Phosphate, mg	931.9 ± 280.1	927.5 ± 192.7	0.63
Iron, mg	11.1 ± 7.4	9.5 ± 2.3	0.68
Zinc, mg	9.7 ± 3.0	8.5 ± 2.2	0.30
Copper, mg	58.0 ± 42.8	106.9 ± 79.2	0.01 *
Alcohol, g	0.0 ± 0.0	1.8 ± 4.6	0.10

^1^ MCI, mild cognitive impairment; DRI, dietary reference intake; M, male; F, female; CHO, carbohydrate; MUFAs, monounsaturated fatty acids; PUFAs, polyunsaturated fatty acids; FAs, fatty acids; SFAs, saturated fatty acids; RE, retinal equivalent. ^2^ Data are expressed as the mean ± standard deviation. ^3^ Statistical analyses were conducted using Student’s *t*-test or Wilcoxon rank-sum test. An asterisk (*) indicates a significant difference compared to the healthy group (*p* < 0.05).

**Table 3:** Dietary intake of six food groups in each group ^1,2,3^.

Food component	Health (*n* = 19)	MCI (*n* = 21)	*p*
Whole grains (servings)	9.4 ± 2.1	9.7 ± 2.1	0.89
Soybeans, fish, eggs, and low-fat meat (servings)	2.8 ± 1.2	1.8 ± 1.1	0.03 *
Soybeans, fish, eggs, and medium-fat meat (servings)	2.6 ± 1.3	3.3 ± 1.5	0.23
Soybeans, fish, eggs, and high-fat meat (servings)	0.1 ± 0.3	0.4 ± 0.6	0.18
Soybeans, fish, eggs, and super high-fat meat (servings)	0.6 ± 0.8	0.5 ± 0.6	0.70
Vegetables (servings)	1.1 ± 0.8	1.4 ± 1.0	0.51
Fruit (servings)	2.1 ± 1.0	2.6 ± 0.8	0.15
Oils, fats, nuts, and seeds (servings)	7.8 ± 3.6	6.5 ± 2.5	0.33
Dairy products (low fat) (servings)	0.1 ± 0.2	0.1 ± 0.2	0.84
Dairy products (whole fat) (servings)	0.3 ± 0.3	0.2 ± 0.3	0.61

^1^ MCI, mild cognitive impairment.^2^ Data are expressed as the mean ± standard deviation.^3^ Statistical analyses were conducted using Student’s *t*-test or the Wilcoxon rank-sum test. * *p* value of <0.05 was statistically significant.

**Table 4:** MIND diet score in each group ^1,2,3^.

	Healthy (*n* = 19)	MCI (*n* = 21)	*p*
Green leafy vegetables score	0.8 ± 0.3	0.8 ± 0.2	1.00
Other vegetables score	0.5 ± 0.5	0.6 ± 0.5	0.79
Berries score	0.0 ± 0.0	0.0 ± 0.0	1.00
Nuts score	0.4 ± 0.4	0.4 ± 0.4	1.00
Olive oil score	0.2 ± 0.4	0.4 ± 0.5	0.15
Butter and margarine score	1.0 ± 0.1	1.0 ± 0.0	0.32
Cheese score	0.9 ± 0.2	1.0 ± 0.2	0.32
Whole grains score	0.2 ± 0.3	0.5 ± 0.5	0.06
Fish (not fried) score	0.8 ± 0.4	0.9 ± 0.4	0.59
Soybeans score	0.7 ± 0.4	0.6 ± 0.5	0.78
Poultry (not fried) score	0.6 ± 0.5	0.6 ± 0.5	0.81
Red meat and products score	0.1 ± 0.3	0.2 ± 0.3	0.28
Fast and fried foods score	0.9 ± 0.2	0.9 ± 0.3	0.76
Pastries and sweets score	0.8 ± 0.4	0.7 ± 0.5	0.35
Wine score	0.0 ± 0.0	0.0 ± 0.2	0.37
MIND diet score	8.0 ± 1.5	8.6 ± 1.2	0.17

^1^ MCI, mild cognitive impairment; MIND diet, Mediterranean-DASH intervention for neurodegenerative delay diet. ^2^ The consumption of olive oil was given a score of 1 if the participant indicated that this was the main oil usually used at home; otherwise, a score of 0 was given. For all other diet components, a concordance score of 0, 0.5, or 1 was assigned by summing the frequency of consumption of each food portion associated with that component. All 15 component scores were summed to give the total MIND diet score. The scoring scale and converted servings of USA and Taiwan are shown in Supplementary Table S1. Data are expressed as the mean ± standard deviation. ^3^ Statistical analyses were conducted using Student’s *t*-test or the Wilcoxon rank-sum test.

**Table 5:** Univariate and multivariate regression of nutrients and cognitive function ^1,2,3^.

	MMSE				CDR			
	Univariate		Multivariate		Univariate		Multivariate	
	β	*p*	β	*p*	β	*p*	β	*p*
Dietary intake levels								
Energy, kcal	<0.001	0.10	−0.09	0.36	<0.001	0.85	−0.01	0.47
CHO, g	0.02	0.06	0.02	0.16	<0.001	0.59	0.04	0.43
Protein, g	0.02	0.22	0.47	0.31	<−0.001	0.96	0.03	0.63
Fat, g	0.01	0.41	0.39	0.67	<−0.001	0.87	0.07	0.5
MUFAs, g	0.03	0.29	0.57	0.21	<0.001	0.88	0.01	0.88
PUFAs, g	<0.001	0.93	−6.50	0.44	−0.01	0.20	−0.11	0.22
n-3 PUFAs, g	−0.05	0.81	6.56	0.44	−0.06	0.04 *	−0.01	0.64
n-6 PUFAs, g	<0.001	0.88	6.87	0.43	<−0.001	0.32	0.13	0.18
SFAs, g	0.03	0.51	0.30	0.40	<0.001	0.60	0.02	0.5
Cholesterol, mg	<0.001	0.18	0.01	0.59	<0.001	0.08	<0.001	0.42
Fiber, g	0.05	0.35	0.17	0.61	<0.001	0.54	0.03	0.46
Vitamins								
Vitamin A, RE	<0.001	0.02 *	<0.001	0.71	<0.001	0.10	<0.001	0.87
Vitamin E, mg	0.02	0.11	0.14	0.13	<0.001	0.11	−0.004	0.46
Vitamin C, mg	0.01	0.008 *	0.01	0.07	<−0.001	0.99	−0.0009	0.68
Vitamin B_1_, mg	0.75	0.16	1.10	0.73	0.09	0.29	0.63	0.12
Vitamin B_2_, mg	1.64	0.06	0.71	0.58	0.05	0.73	0.26	0.71
Niacin, mg	0.08	0.31	−0.01	0.95	0.01	0.31	0.05	0.08
Vitamin B_6_, mg	0.86	0.16	0.79	0.73	−0.01	0.93	−0.7	0.05
Vitamin B_12_, μg	−0.01	0.82	0.01	0.98	−0.01	0.09	<0.001	0.88
Folic acid, μg	<0.001	0.08	−0.006	0.18	<0.001	0.34	−0.0007	0.71
Minerals								
Sodium, mg	<0.001	0.56	<0.001	0.65	<−0.001	0.21	−0.0001	0.82
Potassium, mg	<0.001	0.10	−0.004	0.25	<−0.001	0.80	<0.001	0.58
Calcium, mg	<0.001	0.004 *	0.002	0.04 *	<−0.001	0.73	−0.0009	0.15
Magnesium, mg	<0.001	0.17	0.02	0.58	<−0.001	0.72	−0.002	0.61
Phosphate, mg	<0.001	0.48	−0.01	0.54	<−0.001	0.95	<0.001	0.88
Iron, mg	0.07	0.16	0.12	0.77	−0.01	0.35	0.04	0.49
Zinc, mg	0.12	0.23	−0.51	0.44	−0.02	0.18	−0.04	0.54
Copper, mg	<0.001	0.24	−0.05	0.13	<0.001	0.02 *	0.002	0.02 *
Alcohol, equivalent	0.04	0.59	0.27	0.31	0.02	0.10	0.05	0.17

^1^ MMSE, mini-mental state examination; CDR, clinical dementia rating scale; CHO, carbohydrate; MUFAs, monounsaturated fatty acids; PUFAs, polyunsaturated fatty acids; FAs, fatty acids; SFAs, saturated fatty acids. ^2^ Data were adjusted for gender, age, DM, HTN, and calorie intake. ^3^ Data were adjusted for gender and age. * *p* < 0.05 was statistically significant.

**Table 6:** Univariate and multivariate regression of food components and cognitive function ^1,2^.

	MMSE				CDR			
	Univariate		Multivariate		Univariate		Multivariate	
	β	*p*	β	*p*	β	*p*	β	*p*
Food component								
Whole grains (ex)	0.02	0.86	0.19	0.26	0.01	0.61	−0.02	0.55
Soybeans, fish, eggs, and meat (low fat) (ex)	−0.08	0.73	−0.11	0.7	−0.08	0.01 *	−0.07	0.03 *
Soybeans, fish, eggs, and meat (medium fat) (ex)	0.12	0.52	−0.01	0.96	0.04	0.15	0.05	0.17
Soybeans, fish, eggs, and meat (high fat) (ex)	0.07	0.90	−0.16	0.83	0.14	0.12	0.15	0.16
Soybeans, fish, eggs, and meat (super high fat) (ex)	−0.18	0.65	−0.24	0.62	−0.02	0.68	0.02	0.77
Vegetables (ex)	0.01	0.97	0.15	0.69	0.07	0.14	0.04	0.45
Fruit (ex)	0.76	0.01 *	0.73	0.01 *	0.04	0.33	0.03	0.59
Oils, fats, nuts, and seeds (ex)	0.09	0.30	0.15	0.18	−0.02	0.21	−0.03	0.06
Dairy products (low fat) (ex)	−0.38	0.76	0.21	0.89	0.10	0.62	0.08	0.71
Dairy products (whole fat) (ex)	1.26	0.12	1.75	0.07	−0.05	0.68	0.01	0.96

^1^ MMSE, mini-mental state examination; CDR, clinical dementia rating scale; ex, exchange. ^2^ Data were adjusted for gender, age, DM, HTN, and calorie intake. * *p* < 0.05 was statistically significant.

**Table 7:** Odds ratios (ORs) for mild cognitive impairment (MCI) with MIND diet ^1,2,3,4,5^.

	Low-MIND Diet Score	High-MIND Diet Score	
	OR (95% CI)	OR (95% CI)	*p*
MCI	1 (Reference)	0.20 (0.04–0.99)	0.04 *

^1^ MIND diet, Mediterranean-DASH intervention for neurodegenerative delay diet; CI, confidence interval. ^2^ The high-MIND diet group was defined as MIND score more than the median (>7.5 points). ^3^ The low-MIND diet group was defined as the reference group. ^4^ Average scores for the high-MIND diet group and low-MIND diet group were 9.2 and 6.9. ^5^ Data were adjusted for gender, age, DM, hypertension, and calorie intake. * *p* < 0.05 was statistically significant.

**Table 8:** Blood biochemical analysis in each group ^1,2,3^.

	All (*n* = 40)	Healthy (*n* = 19)	MCI (*n* = 21)	*p*
BUN, mg/dL	17.2 ± 6.7	17.0 ± 8.1	17.3 ± 5.3	0.46
Serum Cr, mg/dL	0.8 ± 0.3	0.8 ± 0.4	0.8 ± 0.3	0.69
Albumin, mg/dL	4.4 ± 0.3	4.4 ± 0.2	4.3 ± 0.4	1.00
FPG, mg/dL	104.6 ± 26.3	107.6 ± 34.2	102.1 ± 18.2	0.86
HbA1c, %	6.2 ± 0.9	6.4 ± 1.3	6.1 ± 0.6	0.89
TC, mg/dL	181.3 ± 34.9	177.4 ± 28.7	184.1 ± 39.3	0.72
HDL-C, mg/dL	54.9 ± 13.1	52.8 ± 12.9	56.5 ± 13.4	0.37
LDL-C, mg/dL	107.0 ± 30.7	104.7 ± 26.1	108.9 ± 34.7	0.88
TGs, mg/dL	104.6 ± 24.9	110.6 ± 30.2	100.4 ± 20.3	0.44
Folate	14.2 ± 8.0	13.0 ± 7.7	15.3 ± 8.3	0.33
Vitamin B_12_	857.5 ± 438.8	743.1 ± 406.1	966.2 ± 451.0	0.08

^1^ MCI, mild cognitive impairment; BUN, blood urea nitrogen; Cr, creatinine; FPG, fasting plasma glucose; HbA1c, glycated hemoglobin; TC, total cholesterol; LDL-C, low-density lipoprotein cholesterol; HDL-C, high-density lipoprotein cholesterol; TGs, triglycerides. ^2^ Data are expressed as the mean ± standard deviation. ^3^ Statistical analyses were conducted using Student’s *t*-test or the Wilcoxon rank-sum test.

## Data Availability

Data regarding investigated patients and analytic methods will be made available on request.

## Supplementary Materials

The following supporting information can be downloaded at https://www.mdpi.com/article/10.3390/nu15173694/s1. Supplementary Table S1. MIND diet component, servings, and scores.
