# Supplementary material for: Evaluation of Dietary Intake in Individuals with Mild Cognitive Impairment
Source: Nutrients. 2023 Aug 23;15(17):3694. doi: 10.3390/nu15173694 (PMC10490258; doi:10.3390/nu15173694)
Supplement: Supplementary file 1 [file nutrients-15-03694-s001.zip › nutrients-2522528-supplementary.pdf]

Supplementary Table S1. MIND diet component, servings, and scores [a]

Item/Score	0	0.5	1
Green leafy vegetables [b]	≤2 servings/wk	> 2 to <6/wk	≥6 servings/wk
Other vegetables [c]	<5 serving/wk	5 to <7 wk	≥1 serving/day
Berries [d]	<1 serving/wk	1 /wk	≥2 servings/wk
Nuts	<1 serving /mo	1/mo to <5/wk	≥5 servings/wk
Olive oil	Not primary oil		Primary oil used
Butter, margarine	>2 times/d	1–2 times/d	<1 times/d
Cheese	7+ servings/wk	1–6 /wk	< 1 serving/wk
Whole grains	<1 serving/d	1–2 /d	≥3 servings/d
Fish (not fried) [e]	Rarely	1–3 /mo	≥1 meals/wk
Beans [f]	<1 meal/wk	1–3/wk	>3 meals/wk
Poultry (not fried) [g]	<1 meal/wk	1 /wk	≥2 meals/wk
Red meat and products [h]	7+ meals/wk	4–6 /wk	< 4 meals/wk
Fast fried foods [i]	4+ times/wk	1–3 /wk	<1 time/wk
Pastries & sweets [j]	7+ servings/wk	5 −6 /wk	<5 servings/wk
Wine	>1 glass/d or never	1/mo to 6/wk	1 glass/d
Total score		15	

[a] Vegetables 1 serving = 0.5 cup, 118.3 g (USA) = 0.5 bowl, 100 g (Taiwan); Berries 1 serving = 0.5 cup, 118.3 g (USA) strawberries 170 g (Taiwan); Nuts 1 serving =1 oz, 28.35 g (USA) = 1 time, 15 g (Taiwan); Grains 1 serving = dry weight 15~20 g (USA) = dry weight 20 g (Taiwan).

[b] Kale, collards, greens; spinach; lettuce/tossed salad.

[c] Green/red peppers, squash, cooked carrots, raw carrots, broccoli, celery, potatoes, peas or lima beans, potatoes, tomatoes, tomato sauce, string beans, beets, corn, zucchini/summer squash/eggplant, coleslaw, potato salad.

[d] Strawberries.

[e] Tuna sandwich, fresh fish as a main dish; not fried fish cakes, sticks, or sandwiches.

[f] Beans, lentils, soybeans.

[g] Chicken or turkey sandwich, chicken or turkey as a main dish and never eat fried at home or away from home.

[h] Cheeseburger, hamburger, beef tacos/burritos, hot dogs/sausages, roast beef or ham sandwich, salami, bologna, or other deli meat sandwich, beef (steak, roast) or lamb as a main dish, pork or ham as a main dish, meat balls or meatloaf.

[i] How often do you eat fried food away from home (like French fries, chicken nuggets)?

[j] Biscuits/rolls, pop tarts, cake, snack cakes/Twinkies, Danish/sweet rolls/pastry, donuts, cookies, brownies, pie, candy bars, other candy, ice cream, pudding, milkshakes/frappes.

MIND, Mediterranean-DASH intervention for neurodegenerative delay.
